# A model-based opinion dynamics approach to tackle vaccine hesitancy

**DOI:** 10.1038/s41598-022-15082-0

**Published:** 2022-07-12

**Authors:** Camilla Ancona, Francesco Lo Iudice, Franco Garofalo, Pietro De Lellis

**Affiliations:** 1 Department of Electrical Engineering and Information Technology, University of Naples Federico II, Naples, 80125 Italy; 2 Department of Management, Information and Production Engineering, University of Bergamo, Bergamo, 24127 Italy

**Keywords:** Complex networks, Health policy

## Abstract

Uncovering the mechanisms underlying the diffusion of vaccine hesitancy is crucial in fighting epidemic spreading. Toward this ambitious goal, we treat vaccine hesitancy as an opinion, whose diffusion in a social group can be shaped over time by the influence of personal beliefs, social pressure, and other exogenous actions, such as pro-vaccine campaigns. We propose a simple mathematical model that, calibrated on survey data, can predict the modification of the pre-existing individual willingness to be vaccinated and estimate the fraction of a population that is expected to adhere to an immunization program. This work paves the way for enabling tools from network control towards the simulation of different intervention plans and the design of more effective targeted pro-vaccine campaigns. Compared to traditional mass media alternatives, these model-based campaigns can exploit the structural properties of social networks to provide a potentially pivotal advantage in epidemic mitigation.

## Introduction

The ongoing COVID-19 pandemic has put the phenomenon of vaccine hesitancy back under the spotlight for the subsequent delays in our race to stem the transmission of the virus^1–3^. Prior to this global emergency, the reluctance that a fraction of the population has in getting vaccinated already proved to be a global threat for human health, see the recent resurgence of measles both in Europe and the US^4–6^. Since the first vaccines were developed, a hesitant attitude in a relevant fraction of the population has been constantly observed at every latitude and across all socio-economic classes^7–9^. Public concerns about vaccines can potentially resonate on social platforms, triggering skepticism towards a recommended vaccination, which in turn translates into delaying or refusing to take the jab. The spectrum of hesitants ranges from fierce antivax, to people who accept vaccines but still remain uncertain about their use. In this social environment, being the vaccination based on voluntary compliance, the fear is that some people might play a wait-and-see game, whereby individuals who choose to wait enjoy the benefits generated by those who do opt for vaccination. This triggers a collective threat that has been highlighted through game theory: rational vaccination decisions based on individual self-interest bring to vaccination levels that are below the optimum for the community^10^. However, rational arguments are seldom at the basis of vaccine hesitancy, which is typically amplified by the rumors spreading on social media^11^. Indeed, opinion formation is not only affected by the social pressure exerted through traditional media outlets such as newspapers or tv, but also by peer-to-peer interactions on social networks. The latter should then probably be the main means for effective promotion campaigns aimed at diffusing the vaccine literacy and boosting immunization acceptance^12^.

An incisive campaign to promote vaccination over a social network requires a suitable selection of the target subjects, and should be tailored to the specific concerns they have on vaccination. Doing so demands the contribution of diverse scientific communities. The large literature on behavioral motivation in medical and social sciences is a precious source of effective communication strategies and arguments to tackle any kind of concern^13–16^. Artificial intelligence and data science may help detect misinformation flowing on social platforms and assess the public confidence in vaccination, see^17^ and references therein. In this context, the contribution of network control^18–20^ could be crucial, since model-based approach may enable the simulation of what-if scenarios corresponding to different promotion campaigns. Here, we show that a network model of opinion diffusion can (i) capture the dynamics of vaccine hesitancy in large groups of individuals and (ii) inform the design of pro-vaccine social media campaigns targeting select individuals within these groups.

Most of the existing models of opinion dynamics have an explanatory character and derive the basic mechanisms of social influence from analogies with diffusion processes in physical systems. Different rules for updating the opinions in the group have been considered, which include imitation^21,22^, averaging over people with similar opinions^23–25^, following the majority^26,27^, and cooperative versus competitive interactions^28–31^. Feeding the huge amount of data that artificial intelligence and data science can mine from social networks to an opinion dynamics model can help unleash the predictive power of these explanatory models under external stimuli, thereby enabling proactive interventions^32^. This paper tries to make a first step in this direction, bridging opinion dynamics and vaccine willingness in a scaled model, which is calibrated on a survey conducted on a sample of the Italian population. This enables us to hypothesize different targeted vaccine promotion campaigns and compare their effectiveness on the basis of the expected fraction of the population that, subject to the each different campaign, will decide to take the vaccine.

## Results

*A dynamic model of vaccine willingness* Vaccine hesitant individuals are defined by WHO as “a heterogeneous group that are indecisive in varying degrees about specific vaccines or vaccination in general”. Hence, vaccine willingness is a “fluid” opinion on vaccination that can be molded by social interaction and external stimuli. Our modelling assumption is that the vaccine willingness of the *i*-th of a population of *n* networked individuals, $x_i(k)$, is shaped in time by social interactions according to the Friedkin-Johnsen model^33^, i.e.,

1 $$\begin{aligned} x_i(k+1)=\lambda _i\sum _{j\in \mathcal {N}_i}w_{ij}x_j(k)+(1-\lambda _i)x_i(0). \end{aligned}$$

Here, the so-called *susceptibility* $\lambda _i\in [0,1]$ modulates the convex combination between agent *i*’s innate opinion $x_i(0)$ and the social pressure modeled as the average of the current willingnesses $x_j(k)$ of its neighbors in the network (the agents in the set $\mathcal {N}_i$). The complement to 1 of $\lambda _i$ captures the agent’s *stubbornness*.

Departing from the consideration that radical views generally translate into foreseeable (unsurprising) actions, while actions related to moderate opinions are far more uncertain, we posit that the probability of an individual accepting a jab at a certain time *k*, $p_i(k)$, depends linearly on its willingness $x_i(k)$. Hence, we can extend the model of $x_i(k)$ to $p_i(k)$ obtaining

2 $$\begin{aligned} p_i(k+1)=\lambda _i\sum _{j\in \mathcal {N}_i}w_{ij}p_j(k)+(1-\lambda _i)p_i(0). \end{aligned}$$

According to our model, the binary decision of taking or refusing a jab becomes a Bernoulli random variable whose parameter is $p_i(k)$.

*Incorporating pro-vaccine campaigns into the model* Exploiting tools from network control^34,35^, we incorporate a pro-vaccine campaign in model (1)–(2) as an additional virtual node, an *influencer agent*, whose willingness is $x_l(k)$ and associated probability of accepting a jab $p_l(k)$. The influencer agent is connected through a directed link to a fraction $\phi$ of targeted individuals. Hence, the dynamics of the targeted agents becomes

3 $$\begin{aligned} p_i(k+1)=\Big ((1-\alpha )\lambda _i \sum _{j\in \mathcal {N}_i} p_j(k)+\alpha p_l(k) \Big ) + (1-\lambda _i) p_i(0), \end{aligned}$$

where $\alpha \in [0,1]$ quantifies the *effort per target individual*. Hence, we characterize the *overall effort* $0\le \eta \le 1$ of a campaign as the product of the two parameters $\alpha$ and $\phi$. During the ongoing pandemic, health authorities of most countries have conducted traditional pro-vaccine campaigns through mass media to fight vaccine hesitancy^36–38^. In our modeling framework, this means that the influencer (in this case, the health authority) is connected to all the network agents, that is, $\phi =1$. However, in the era of online social media and targeted marketing, one could argue that a targeted pro-vaccine campaign, where the influencer devotes a larger individual effort $\alpha$ to a small fraction $\phi$ of the agents, could outperform traditional mass campaigns given the same overall effort $\eta$.

To dispel this doubt, we exploit our scaled model to design three alternative targeted campaigns, differing for the selection of the targeted agents, denoted in the following as Strategies 1, 2, and 3, respectively. Strategy 1, as in classical network science approaches, targets the most connected agents, i.e. the agents that have the greatest topological advantage for spreading opinions favourable to vaccination. Strategy 2 mitigates the effect of the antivax by targeting their neighbors, whereas Strategy 3 directly targets the most susceptible agents. The details on the implementation of these campaigns are provided in the Methods.

It is worth pointing out that the three strategies we propose require different information levels, thus posing different feasibility issues. Indeed, targeting the most connected agents only requires knowledge of the unweighted topology of the social network. Attempting instead at neutralizing the antivax requires to complement this structural information with that on the agents’ vaccine hesitancy, which can be monitored by means e.g. of sentiment analysis on social media^39,40^. Finally, directly influencing the most susceptible agents constitutes a psychological targeting strategy (see^41^ and the references therein for alternatives methods to do so) that requires assessing the personality traits of each individual.

*A scaled model of vaccine willingness in the Italian population* We exploit our modeling framework to build a scaled representation of vaccine willingness in the Italian population. Since we focus on interactions taking place through *online* social media, we borrowed the graph describing social interactions among the individuals from a Facebook friendship network^42^. We associate to the individuals of our scaled model vaccine willingnesses whose distribution is compatible with the outcome of a survey conducted on a sample of the Italian adult population at the end of the first lockdown^8^, when the vaccine availability was long to come. From these data we were able to estimate the susceptibilities $\lambda _i$ so as to preserve, at steady-state, the aforementioned association, see the Methods for details and the Supplementary Information, Figure S7, for a graphical representation. The steady-state distribution of the vaccine willingness enables the evaluation of the probability that any given fraction of the population gets vaccinated (see Section S1 of the Supplementary Information), which in turn allows to compute the expected fraction of the population that, at the time of the survey, would have taken a jab had this opportunity been given.

**Figure 1:**
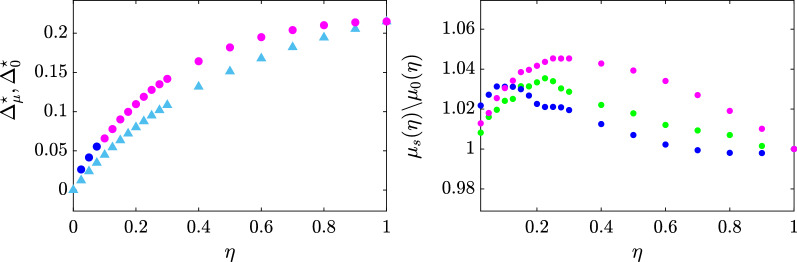
Comparison of targeted and traditional provax mass campaigns. The left panel depicts, for each effort $\eta$, the additional population fraction $\Delta _{\mu }^\star$ and $\Delta _{\mu }^0$ that is expected to be vaccinated when the best targeted campaign (identified by circles) or the mass provax campaign (identified by triangles) are employed, respectively. The right panel displays for each effort $\eta$ and targeted strategy *s*, the ratio between the fractions of the population $\mu _s(\eta )$ and $\mu _0(\eta )$ that are expected to be vaccinated when strategy *s* and the traditional campaign are employed, respectively. In both panels, Strategy 1, 2, and 3 are depicted in blue, green, and magenta, respectively, the intensity of the vaccination campaign is set to $\alpha = 1$ and for the maximum effort $\eta = 1$, all points are superimposed since all strategies would be equivalent.

*Comparing pro-vaccine campaigns* Leveraging our scaled model, we conducted a numerical analysis to compare the effectiveness of targeted and mass campaigns on our synthetic population. Our simulations show that i) the targeted campaigns outperform a general mass-media campaign, and ii) the best strategy for targeting individuals depends on the overall effort $\eta$ of the campaign. Indeed, for all possible selections of $\eta$, it is possible to find a targeted strategy that yields an advantage compared with general mass-media campaigns, with an increase of the expected number of vaccinated individuals that reaches a maximum $5\%$ for $\eta =0.25$, see the left panel of Fig. 1. Interestingly, for low efforts ($\eta <0.1$), any strategy is capable of increasing the effectiveness of the vaccination campaign, with the merely topological approach of Strategy 1 being the most effective. When more resources can be devoted to the campaign, our model predicts that a finer characterization of the individuals is required to substantially increase the expected vaccinated population, see the right panel of Fig. 1. In particular, for all $\eta \ge 0.1$, Strategy 3, which relies on the estimation of the individual susceptibility, proves to be the best campaign. One could argue that the expected advantage of targeted strategies over the general alternative could be irrelevant, should the variance be high. However, as shown in the Supplementary Information, the variance of the distribution of the fraction of vaccinated individuals tends to 0 as the size of the populations increases, and is negligible when we consider the population of a country like Italy. These results are robust to changing the graph underlying our scaled model, see Section S3 of the Supplementary Information.

**Figure 2:**
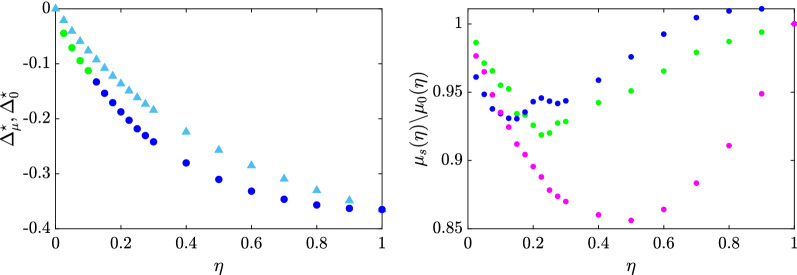
Comparison of the targeted and traditional mass antivax campaigns. The left panel depicts, for each effort $\eta$, the additional population fraction $\Delta _{\mu }^\star$ and $\Delta _{\mu }^0$ that is expected to be vaccinated when the best targeted (identified by circles) or the mass (identified by triangles) antivax campaigns are employed, respectively. The right panel displays for each effort $\eta$ and targeted strategy *s*, the ratio between the fractions of the population $\mu _s(\eta )$ and $\mu _0(\eta )$ that are expected to be vaccinated when strategy *s* and the mass antivax campaign are employed, respectively. In both panels, Strategy 1, 2, and 3 are depicted in blue, green, and magenta, respectively, the intensity of the vaccination campaign is set to $\alpha = 1$, and, for the maximum effort $\eta = 1$, all points are superimposed since all strategies would be equivalent.

*Impact of antivax campaigns* Our model can also be used to assess the possible impact of antivax campaigns. Analogously to the provax case, we incorporate the role of antivax campaigns attempting to polarize the vaccination probabilities towards zero by setting $p_l(k)=0$ for all *k*. Moreover, we assume that the selection of the agents targeted by the antivax influencer is made according to the same criteria defining the provax strategies. As illustrated in Fig. 2, antivax campaigns can be even more impactful than their provax counterparts and thus can represent a serious hindrance in our quest to stem the transmission of the virus.

## Discussion

In this paper, we proposed a model-based approach, grounded in opinion dynamics, which identifies the patterns through which the vaccine hesitancy/willingness diffuses in a population. The availability of such a model offers, potentially, two major benefits. The first is the possibility of predicting the fraction of a given population that, with a certain confidence level, will decide to get vaccinated, thus enriching the information that can be drawn from the numerous surveys on vaccine willingness. The second and more crucial benefit consists in the possibility of simulating alternative scenarios where different pro-vaccine media campaigns over social media are enacted. Prior to their implementation, the campaigns can then be designed and tested on a scaled model, so that their effectiveness can be maximized.

Our results indicate that targeted campaigns always outperform mass campaigns, yielding the maximum increment in the expected vaccinated population for intermediate values of the overall effort of the campaign. For such values, the gain of electing smart, targeted campaigns rather than mass campaigns is to increase the expected vaccinated population by an additional $5\%$. Implementing targeted campaigns entails the use of tools from artificial intelligence and data science, with higher investments needed compared to traditional campaigns. It is reasonable to ask whether this additional burden is worth carrying: can a marginal increase of vaccinated individuals make a difference? From the perspective of protecting public health, the empirical answer lies in the recent data showing that avoiding saturation of healthcare sys is a matter of slight differences in the number of vaccinated individuals^43^. Modest increases in the effectiveness of a campaign can well be the difference between expecting or not to live with the virus without restrictions. However, from an ethical perspective one could argue that targeting individuals based on information obtained from its online social media might surpass the borders protecting the privacy of the population. Where the optimal trade-off lies between feasibility and ethics is subject of ongoing worldwide discussions^44^.

From a methodological perspective our work represents a first step toward bridging the abstract literature on opinion dynamics with the pressing open problem of fighting vaccine hesitancy. Although the results are promising, our work is not free of limitations, and there are several directions along which it can be extended. First, in its current incarnation, model calibration is only concerned with steady-state vaccination probabilities. This is certainly sufficient when the campaigns are planned way ahead the administration of the vaccine. However, in the case of a new epidemic, news from media outlets may perturb the beliefs of the population, see e.g. the scientific and social debate on the AstraZeneca COVID-19 vaccine^45,46^. In such cases, the campaign should be immediately redesigned, and tailored on the basis of the response time of the population. Our model has the potential to account for these transient dynamics, provided that several snapshots of the opinions of the same cohort of the population are available. Having access to additional snapshots would also allow considering weighted networks. Second, alternative models of opinion dynamics could be considered. In the spectrum of model complexity, we decided to opt for the simplest one, so as to minimize the number of parameters to be tuned. Should one have more data for finer calibrations, alternative, more complex models of opinion dynamics could be considered to account e.g. for bounded confidence^24^, or for the difference between private and publicly expressed opinions^22^. Third, our assumption that vaccination decisions were grounded on steady-state willingness was justified by the fact that the COVID-19 pandemic was characterized by a one year delay between the first prospect and mass availability of vaccines. As this could not be the case in the future, it could be interesting to evaluate the effectiveness of provax campaigns when removing this assumption. In this vein, the model could be reworked so to consider the influence of an individual’s decision on the willingness of its peers. Finally, since it has been observed that social networks may be characterized by the presence of communities of like-minded individuals^47^, which are socially well connected and share many interests, an open research question is to evaluate how these densely connected communities may affects the effectiveness of targeted vaccination campaigns.

The reductive choice of characterizing the behavior or each individual through the Friedkin-Johnsen model allows for a first assessment of the effectiveness of pro-vaccine campaigns on the basis of data collected from a single survey. Indeed, the strength of our inherently causal model-based approach lies in the ability of teasing out the relationship between the choice of the targets of the campaign and its effectiveness. This ease of interpretation is a feature we believe should be retained even when more refined data on vaccine hesitancy are considered.

## Methods

### Opinion dynamics modeling of vaccine acceptance

In model (1), vaccine willingness diffuses along an undirected connected graph $\mathcal {G}=\{\mathcal {V},\mathcal {E}\}$ with self-loops at each node, where $\mathcal {V}$ is the set of the *n* individuals, and $\mathcal {E}=\{(i,j) \subseteq \mathcal {V} \times \mathcal {V} \}$ is the set of edges connecting neighboring individuals. We posit that an individual’s probability of accepting a jab is linearly proportional to its vaccine willingess leading to Eq. (2), that can be rewritten in compact matrix form as

4 $$\begin{aligned} p(k+1) = \Lambda Wp(k) + (I_n - \Lambda ) p(0), \end{aligned}$$

where $\Lambda =\mathrm {diag}\{[\lambda _1,\ldots ,\lambda _n]^\mathrm {T}\}$ encodes the susceptibilities of each individual and $I_n$ is the identity matrix of size *n*. Moreover, *W* is a row-stochastic matrix that captures the structure of the graph $\mathcal {G}$, whereby its *ij*-th entry $w_{ij}$ is $w_{ij}=1/| \mathcal {N}_i|$ if $(i,j)\in \mathcal {E}$ and zero otherwise, with $| \cdot |$ denoting the cardinality of a set. Finally, *p*(0) encodes the initial willingness of being vaccinated. Note that $\lambda _i=0$ corresponds to a *zealot*^48,49^, who never changes its opinion while actively trying to convince the others. Assuming that $\Lambda \ne I_n$, that is, there exists at least an agent *i* such that $\lambda _i<1$, the vaccination probabilities will converge at steady-state toward^50^

5 $$\begin{aligned} \overline{p}=(I_n-\Lambda W)^{-1}(I_n-\Lambda )p(0), \end{aligned}$$

where $\overline{p}=[\overline{p}_1,\ldots ,\overline{p}_n]^\mathrm {T}$. Knowing the distribution of all individual vaccination probabilities *p*(*k*) allows computing the probability that, at time *k*, a given fraction of the population is willing to be vaccinated. Indeed, this event can be viewed as the outcome of a Poisson binomial experiment, which is a collection of *n* independent yes/no experiments with success probabilities $p_1(k),\ldots ,p_n(k)$. The same consideration holds for the steady-state distribution $\overline{p}$.

### Tuning the model parameters on real data

The parameters that need to be selected in model (4) are related to i) how individuals are connected, which is encapsulated by the network topology, described by matrix *W*, and ii) the inherent characteristics of each individual, captured by the susceptibilities $\lambda _1,\ldots ,\lambda _n$, and by the initial probabilities $p_1(0),\ldots ,p_n(0)$, a measure of their pre-existing attitude towards vaccines. The network matrix *W* has been borrowed from a Facebook social friendship network^42^, composed by $n=1446$ nodes, with $|\mathcal {E}|=59600$ edges describing their mutual interactions. We have chosen the individual parameters so that the steady-state probabilities $\overline{p}$ in (5) are compatible with the outcome of a survey administered to a sample of Italian citizens^8^. Toward this goal, we first translated the survey outcome into target steady-state values $p^\star$, to then tune the susceptibilities $\lambda _i$ and find a set of initial attitudes $p_i(0)$ so to obtain the $\overline{p}$ that best matches $p^\star$ in the least square sense (see Figure S7 of the Supplementary Information for a visualization of $\overline{p}$ and $p^\star$).

#### Description of the dataset from^8^ and choice of $p^\star$

The authors of^8^ tested the beliefs and attitudes of Italian citizens towards a possible COVID-19 vaccine through the administration of surveys, based on the Likert scale, to a stratified sample of 1004 individuals, representative of the Italian adult population aged between 18 and 70 years old. The respondents filled the survey during the first days following the end of Italy’s strict lockdown begun in March 2020, when no vaccine was available yet. The survey contained general questions about their lives and health habits, as well as specific questions related to the COVID-19 pandemic. In this work, we focused on the 5th Likert item of the survey, which reads *‘I am willing to vaccinate, if a vaccine against COVID-19 were to be found’*, with five options, ranging from $1 =$ *‘not likely at all’* to $5 =$ *‘absolutely’*, and computed the fraction $f_j$ of agents choosing answer *j* to question 5, for $j=1,\ldots ,5$.

Accordingly, we partitioned our social network of $n=1446$ nodes into 5 classes, where the *j*-th class is populated by the $c_j= f_j n$ agents expected to choose option *i*. As $f_j n$ is not necessarily an integer, it is rounded so that $\sum _{i=1}^5 c_j =n$, and each agent is randomly assigned to each class. We then converted the categorical values of the Likert scale into continuous values in the interval [0, 1] following the approach in^51^, and splitting it in 5 sub-intervals, one for each class (alternative approaches have been proposed e.g. in^52,53^). Namely, the *j*th class was associated to a range $r_j=[0.2(j-1),0.2 j]$, $j=1,\ldots ,5$, where the steady-state vaccination probabilities $p^{\star }$ should lie, see Table 1. Given an agent *i* assigned to class *j*, the steady-state vaccination probability $p_i^{\star }$ has been extracted from a uniform distribution in $r_j$.

**Table 1:** Conversion of discrete vaccine willingness Likert score to continuous probability of getting vaccinated.

Likert item point	Probability range $r_j$
(1) Not likely at all.	0 – 0.2
(2) A little likely.	0.2 – 0.4
(3) Not likely nor unlikely.	0.4 – 0.6
(4) Very likely.	0.6 – 0.8
(5) Absolutely.	0.8 – 1

#### Selection of the behavioral parameters $\lambda$ and *p*(0)

Once we generated target steady-state probabilities $p^\star$ as explained above, we selected the individual parameters in our network so that the network dynamics converge to the steady-state vaccination probability $\overline{p}$ that is the closest possible to $p^\star$ in the least square sense. Namely,

6a $$\begin{aligned}&\underset{\lambda ,p(0)}{\min } \left\| \overline{p} - p^\star \right\| ^2 \end{aligned}$$

6b $$\begin{aligned}&\mathrm {subject \ to}\nonumber \\&0\le p_i(0) \le 1,\qquad \qquad \quad \ \ \ \, i=1,\ldots ,n, \end{aligned}$$

6c $$\begin{aligned}&\lambda ^\mathrm {T}\mathbbm {1}_n =\rho n,\quad 0\le \lambda _i\le 1,\quad i=1,\ldots ,n, \end{aligned}$$

6d $$\begin{aligned}&\frac{\lfloor 5 p^\star _i\rfloor }{5} < \overline{p}_i\le \frac{\lceil 5 p^\star _i\rceil }{5},\qquad \quad \, i=1,\ldots ,n, \end{aligned}$$

6e $$\begin{aligned}&\overline{p}=(I_n-\Lambda W)^{-1}(I_n-\Lambda )p(0), \end{aligned}$$

where $\lfloor \cdot \rfloor$ and $\lceil \cdot \rceil$ map a real number to its previous or next integer, respectively, and $\mathbbm {1}_n$ is the vector of all ones. Notice that the set of enforced constraints (6b)–(6e) guarantee that the outcome of the optimization is meaningful. Indeed, constraint (6b) guarantees that the probabilities lie in [0, 1], (6c) that the average susceptibility to the neighbors’ opinion is $0<\rho <1$ and the individual susceptibilities belong to [0, 1], whereas (6d) enforces that if $p_i^\star \in r_j$, then also $\bar{p}_i\in r_j$, that is, each agents stays in the target class identified by $p^\star$. Finally, constraint (6e) ensures that the steady-state values $\bar{p}$ are compatible with the dynamics (4). In all our numerical analysis, we selected the largest value of $\rho$ for which problem (6) admits a solution, that is, $\rho =0.58$. However, our main results would still hold for lower values of $\rho$, see Supplementary Information S2 for further details.

#### Incorporating the effect of pro-vaccine campaigns

Once the model has been tuned following the steps described above, we used it to test the effect of alternative pro-vaccine campaigns. According to the Friedkin-Johnsen model, the individuals can neither change their own belief nor their susceptibility, thereby in the time-scale of a campaign we can only act on the social interaction term $\lambda W p(k)$ in (4). Specifically, we model the effect of the vaccination campaign on agent *i* as the addition of a virtual neighbor *l* whose probability $p_l$ of being vaccinated is equal to 1 for all *k*. Agent *i* will weigh the opinion of this virtual agent proportionally to the intensity of the vaccination campaign. In formal terms, Eq. (4) modifies as

7 $$\begin{aligned} p(k+1)=\Lambda \Big ( (I_n-\alpha \Delta ) W p(k)+\alpha \delta p_l \Big ) + (I_n-\Lambda ) p(0), \end{aligned}$$

where $\delta =[\delta _1,\ldots ,\delta _n]^\mathrm {T}$, with $\delta _i$ being 1 if node *i* is targeted by the campaign, and 0 otherwise, $\Delta =\mathrm {diag}\{\delta \}$, $0\le \alpha \le 1$ quantifies the intensity of the vaccination campaign, $p_l=1$ is the vaccination probability the campaign is targeting, and we set $p(0)=\bar{p}$. Namely, $\alpha =0$ corresponds to no effect, whereas $\alpha =1$ to the agents disregarding the opinion of the other neighbors, and only considering the that of the virtual neighbor *l*. The same approach can be used to incorporate the effect of hoaxes and misinformation, just by setting $p_l$ to zero.

## Supplementary information

Supplementary Information.

## Data Availability

The survey raw data on vaccine willingness are publicly accessible from the Supplementary Information of^8^ available online. The network topology that we have used as reference in this work is publicly available from the repository^42^ under the name “Socfb-Haverford-76”.

## Code availability

ll code for the model is publicly available on Open Science Framework (OSF) https://osf.io/7ndmx/?view_only=e0afacbe567147b0ad72ee1fce416c45.

## References

[1] Dror AA (2020). Vaccine hesitancy: the next challenge in the fight against COVID-19. Eur. J. Epidemiol..

[2] Aschwanden C (2021). Five reasons why COVID herd immunity is probably impossible. Nature.

[3] Ophir, Y. et al. Vaccine hesitancy under the magnifying glass: A systematic review of the uses and misuses of an increasingly popular construct. Health Commun., 1–15 (2022). 10.1080/10410236.2022.2054102 35361020

[4] Feemster KA, Szipszky C (2020). Resurgence of measles in the United States: how did we get here?. Curr. Opin. Pediatr..

[5] Wilder-Smith AB, Qureshi K (2020). Resurgence of measles in Europe: a systematic review on parental attitudes and beliefs of measles vaccine. J. Epidemiol. Glob. Health.

[6] Dimala CA, Kadia BM, Nji MAM, Bechem NN (2021). Factors associated with measles resurgence in the United States in the post-elimination era. Sci. Rep..

[7] Lazarus JV (2021). A global survey of potential acceptance of a COVID-19 vaccine. Nat. Med..

[8] Graffigna, G., Palamenghi, L., Boccia, S. & Barello, S. Relationship between citizens’ health engagement and intention to take the COVID-19 vaccine in Italy: a mediation analysis. Vaccines **8** (2020). 10.3390/vaccines8040576 PMC7711984 33019663

[9] Peretti-Watel P (2020). A future vaccination campaign against COVID-19 at risk of vaccine hesitancy and politicisation. Lancet. Infect. Dis.

[10] Bauch CT, Earn DJD (2004). Vaccination and the theory of games. Proc. Natl. Acad. Sci..

[11] Islam MS (2021). COVID-19 vaccine rumors and conspiracy theories: The need for cognitive inoculation against misinformation to improve vaccine adherence. PLoS ONE.

[12] Steffens MS, Dunn AG, Leask J, Wiley KE (2020). Using social media for vaccination promotion: Practices and challenges. Digital Health.

[13] MacDonald NE (2015). Vaccine hesitancy: Definition, scope and determinants. Vaccine.

[14] Chou W-YS, Budenz A (2020). Considering Emotion in COVID-19 vaccine communication: addressing vaccine hesitancy and fostering vaccine confidence. Health Commun..

[15] Kestenbaum LA, Feemster KA (2015). Identifying and addressing vaccine hesitancy. Pediatr. Ann..

[16] Amin AB (2017). Association of moral values with vaccine hesitancy. Nat. Hum. Behav..

[17] Hussain, A. & Sheikh, A. Opportunities for artificial intelligence–enabled social media analysis of public attitudes toward Covid-19 vaccines. NEJM Catal. Innov. Care Delivery **2** (2021).

[18] Liu Y-Y, Barabási A-L (2016). Control principles of complex systems. Rev. Mod. Phys..

[19] Lo Iudice, F., Garofalo, F. & Sorrentino, F. Structural permeability of complex networks to control signals. Nat. Commun. **6**, 1–6 (2015). 10.1038/ncomms9349 PMC4595749 26391186

[20] Della Rossa, F. et al. A network model of Italy shows that intermittent regional strategies can alleviate the COVID-19 epidemic. Nat. Commun. **11**, 1–9 (2020). 10.1038/s41467-020-18827-5 PMC7547104 33037190

[21] Garofalo F, LoIudice F, Napoletano E (2018). Herding as a consensus problem. Nonlinear Dyn..

[22] Ye M, Qin Y, Govaert A, Anderson BDO, Cao M (2019). An influence network model to study discrepancies in expressed and private opinions. Automatica.

[23] Weisbuch G (2004). Bounded confidence and social networks. Eur. Phys. J. B.

[24] Hegselmann, R., Krause, U., et al. Opinion dynamics and bounded confidence models, analysis, and simulation. J. Artif. Soc. Soc. Simul. **5** (2002).

[25] Dandekar P, Goel A, Lee DT (2013). Biased assimilation, homophily, and the dynamics of polarization. Proc. Natl. Acad. Sci..

[26] Javarone MA (2014). Social influences in opinion dynamics: the role of conformity. Physica A.

[27] Krapivsky PL, Redner S (2003). Dynamics of majority rule in two-state interacting spin systems. Phys. Rev. Lett..

[28] Bizyaeva, A., Franci, A. & Leonard, N. E. Nonlinear opinion dynamics with tunable sensitivity. IEEE Trans. Autom. Control (2022).

[29] Altafini C (2012). Consensus problems on networks with antagonistic interactions. IEEE Trans. Autom. Control.

[30] Altafini C, Ceragioli F (2018). Signed bounded confidence models for opinion dynamics. Automatica.

[31] Tangredi D, Iervolino R, Vasca F (2017). Consensus stability in the Hegselmann-Krause model with coopetition and cooperosity. IFAC-PapersOnLine.

[32] Hofman, J. M. et al. Integrating explanation and prediction in computational social science. Nature, 1–8 (2021). 10.1038/s41586-021-03659-0 34194044

[33] Friedkin NE, Johnsen EC (1990). Social influence and opinions. J. Math. Sociol..

[34] DeLellis P, Garofalo F, Lo Iudice F (2018). The partial pinning control strategy for large complex networks. Automatica.

[35] Sorrentino F, di Bernardo M, Garofalo F, Chen G (2007). Controllability of complex networks via pinning. Phys. Rev. E.

[36] Guardian, T. https://www.theguardian.com/world/2021/mar/05/covid-vaccine-adsaim-to-influence-without-alienating-people (2021).

[37] Department, A. G. H. https://www.health.gov.au/news/new-information-campaignto-encourage-australians-to-get-a-covid-19-vaccine (2021).

[38] News, N. https://www.nbcnews.com/health/health-news/sweeping-ad-campaignwill-encourage-vaccinations-rcna309 (2021)

[39] Muric G, Wu Y, Ferrara E (2021). COVID-19 vaccine hesitancy on social media: building a public Twitter dataset of anti-vaccine content, vaccine misinformation and conspiracies. JMIR Public Health Surveill..

[40] Piedrahita-Valdés H (2021). Vaccine hesitancy on social media: Sentiment analysis from June 2011 to April 2019. Vaccines.

[41] Kosinski M, Stillwell D, Graepel T (2013). Private traits and attributes are predictable from digital records of human behavior. Proc. Natl. Acad. Sci..

[42] Rossi, R. A. & Ahmed, N. K. The network data repository with interactive graph analytics and visualization in AAAI (2015). http://networkrepository.com.

[43] Trentini F (2022). Pressure on the health-care system and intensive care utilization during the COVID-19 outbreak in the lombardy region of Italy: A retrospective observational study in 43,538 hospitalized patients. Am. J. Epidemiol..

[44] Asadi Someh, I., Breidbach, C. F., Davern, M. & Shanks, G. Ethical implications of big data analytics. Res. Progress Papers **24** (2016).

[45] Østergaard SD, Schmidt M, Horváth-Puhó E, Thomsen RW, Sørensen HT (2021). Thromboembolism and the Oxford-AstraZeneca COVID-19 vaccine: Side-effect or coincidence?. The Lancet.

[46] Larson HJ, Broniatowski DA (2021). Volatility of vaccine confidence. Science.

[47] Modani N (2014). Like-minded communities: bringing the familiarity and similarity together. World Wide Web.

[48] Cardillo, A. & Masuda, N. Critical mass effect in evolutionary games triggered by zealots. *Physical Review Research* **2**, 023305 (2020).

[49] Verma G, Swami A, Chan K (2014). The impact of competing zealots on opinion dynamics. Physica A.

[50] Proskurnikov AV, Tempo R (2017). A tutorial on modeling and analysis of dynamic social networks. Part I.. Ann. Rev. Control.

[51] Polemi, N. in Securing Critical Information Infrastructures and Supply Chains (Elsevier, 2017).

[52] Sullivan GM, Artino AR (2013). Analyzing and interpreting data from Likert-type scales. J. Grad. Med. Educ..

[53] Carifio J, Perla R (2008). Resolving the 50-year debate around using and misusing Likert scales. Med. Educ..

