# Supplementary material for: A model-based opinion dynamics approach to tackle vaccine hesitancy
Source: Sci Rep. 2022 Jul 12;12:11835. doi: 10.1038/s41598-022-15082-0 (PMC9276809; doi:10.1038/s41598-022-15082-0)
Supplement: Supplementary file 1 — Supplementary Information. [file 41598_2022_15082_MOESM1_ESM.pdf]

Supplementary Information

A Model-based Opinion Dynamics Approach to Tackle Vaccine Hesitancy

Camilla Ancona[1,2,†], Francesco Lo Iudice[1,†], Franco Garofalo[1,*], Pietro De Lellis[1,*]

[1] *Departiment of Electrical Engineering and Information Techonology, University of Naples Federico II, Via Claudio 21, 80125 Italy*

[2] *Department of Management, Information and Production Engineering, University of Bergamo, via dei Caniana 2, 24127 Italy*

[†] *These authors contributed equally*

[*] *Corresponding authors: franco.garofalo@unina.it; pietro.delellis@unina.it*

S1 Mean and variance of Y_n as a function of n

The model presented interprets each agent's opinion as its probability p_i of getting vaccinated. We can then associate to each agent $i = 1, \ldots, n$ independent Bernoulli variables $X_1, \ldots, X_n$, with *heterogeneous* probability of success $p_1, \ldots, p_n$, respectively. As our ultimate goal is to estimate the expected *fraction* of the population that will get vaccinated, we define the stochastic variable

$$Y_n = \frac{1}{n}\sum_{i=1}^{n} X_i,$$

whose expected value and variance can be computed as

$$E[Y_n] = \frac{1}{n}\sum_{i=1}^{n} p_i, \quad \mathrm{Var}[Y_n] = \frac{1}{n^2}\sum_{i=1}^{n} p_i(1-p_i), \tag{S1}$$

respectively.

Note that Y_i is a Poisson binomial distribution (scaled by the factor $1/n$), that is the sum of n independent Bernoulli distributions. Here, we study how its moments scale with the population size n. Denoting $n_0 = 1446$ the number of participants to the survey on which the opinion dynamics model is parametrized in the main text, we scale the population as multiples of n_0, so that we can always associate a vaccination probability p_i to a fraction $1/n_0$ of the total population n, for all $i = 1, \ldots, n_0$. Specifically, we introduce the parameter $\beta_k := \lfloor l_k/n_0 \rceil$ with $l_k = 10^k$, and $k = 4, \ldots, 7$. This gimmick allows us to inspect the behaviour of the moments of the Poisson Binomial distribution when the size of the population is $n = \beta_k n_0$. In turn, from equation (S1) this reflects into the following scaling behavior of the first and second moment of Y_n

$$E[Y_n] = \frac{1}{\beta_k n_0} \sum_{i=1}^{n_0} \beta_k \mathrm{E}(X_i) = \frac{1}{n_0} \sum_{i=1}^{n_0} p_i = E[Y_{n_0}]. \tag{S2a}$$

$$\mathrm{Var}[Y_n] = \frac{1}{(\beta_k n_0)^2} \sum_{i=1}^{n_0} \beta_k p_i (1 - p_i) = \frac{1}{\beta_k} \mathrm{Var}[Y_{n_0}]. \tag{S2b}$$

Hence,

- the expected value $\mathrm{E}[Y_n]$ of the fraction of the population that will get a shot of vaccine does not change with the population size;
- the variance $\mathrm{Var}[Y_n]$ decreases linearly with the population size.

Table S1 reports the mean and variance of Y_n for different orders of magnitudes for l_k, whereas Figure S1 the error bar of the fraction of vaccinated population as a function of the population size n.

l_k	β_k	$\mathrm{E}[Y_n]$	$\mathrm{Var}[Y_n]$
10^4	7	0.6171	1.773e-05
10^5	69	0.6171	1.799e-06
10^6	692	0.6171	1.793e-07
10^7	6916	0.6171	1.794e-08

Table S1: First and second moment of Y_n as the population size $n = \beta_k n_0$ varies.

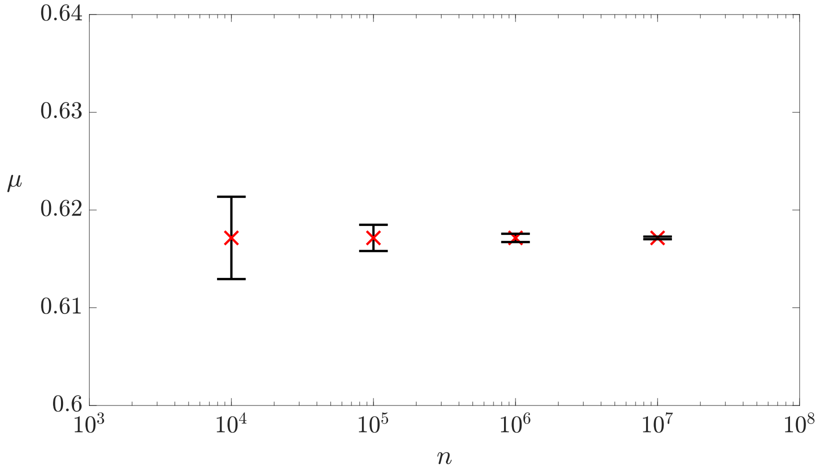

Figure S1: Error bar plot of the mean and standard deviation of Y_n.

S2 Parametric analysis of ρ

In the second subsection of the Methods section of the main text, we presented a constrained least square optimization problem aimed to realistically calibrate the model parameters consistently with survey data. In particular, in the in the main text we explained that constraint (4c) sets the average susceptibility to be equal to a value ρ, and that problem (4) admits a solution only if $\rho \leq 0.58$. In the main text, all the analysis have been performed for $\rho = 0.58$. Here, we perform a parametric analysis of the results whereby we vary ρ in the interval $[0.18, 0.48]$ with step 0.1. Figure S2 illustrates that the results are qualitatively similar, the only difference being the attenuated effectiveness of all the strategies, since lower values of the λ_i correspond to a more stubborn population.

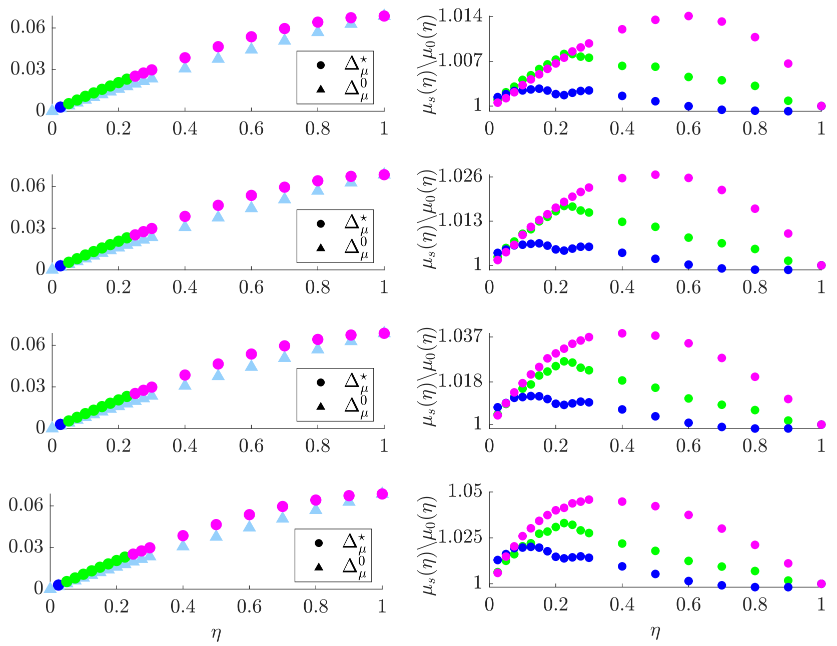

Figure S2: Comparison of targeted and traditional mass campaigns for different values of the average susceptibility ρ (set to $0.18, 0.28, 0.38$, and 0.48, from top to bottom). The left panels depict, for each effort η, the additional population fraction $\Delta_\mu^\star$ and Δ_μ^0 that is expected to be vaccinated when the best targeted campaign (identified by circles) or the mass campaign (identified by triangles) are employed, respectively. The right panels display for each effort η and targeted strategy s, the ratio between the fractions of the population $\mu_s(\eta)$ and $\mu_0(\eta)$ that are expected to be vaccinated when strategy s and the traditional campaign are employed, respectively. In all panels, Strategies 1, 2, 3 are depicted in blue, green, and magenta, respectively, and the intensity of the vaccination campaign is set to $\alpha = 1$.

S3 Robustness analysis

To test the robustness of our results we have run additional simulations varying the graph through which opinions diffuse. To do so, we repeated the same analysis performed in the main text on a pool of real and synthetic networks, whereby we compared the effectiveness of the targeted campaigns to that of mass campaigns. As shown in Figures S3-S6, the results are consistent with the case illustrated in the main text, that is, the targeted campaigns always outperforms the traditional mass-media ones.

The pool of synthetic networks is made of 10 unweighted undirected graphs of size $N = 1500$, extracted from a Scale-Free distribution with exponent $\gamma = 2.8$ and average degree $k_{av} = 80$, consistently with the properties of the real online

social networks reported in [1], and repeated the same analysis performed in the main text. The 3 real networks, called soc-fbHamilton46, socfb-Simmons81 and Hamsterster, have been retrieved from the network repository [1].

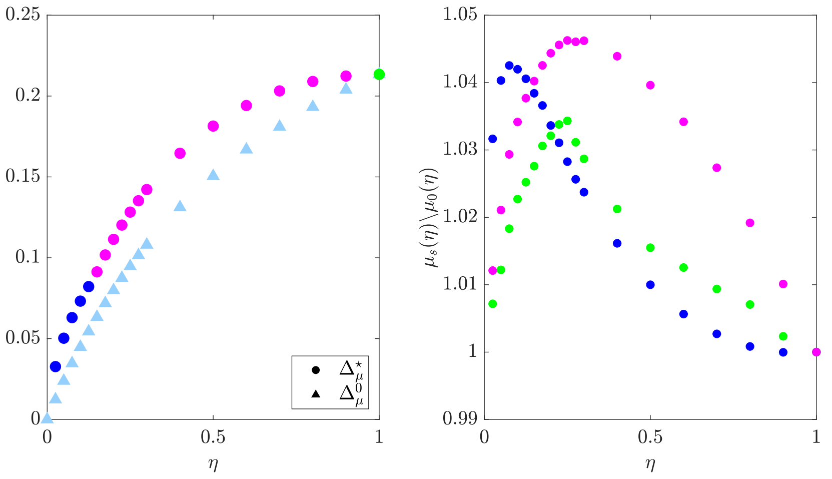

Figure S3: Comparison of targeted and traditional mass campaigns effectiveness averaged on a pool of 10 Scale-Free synthetic networks. The left panels depict, for each effort η, the additional population fraction $\Delta_\mu^\star$ and Δ_μ^0 that is expected to be vaccinated when the best targeted campaign (identified by circles) or the mass campaign (identified by triangles) are employed, respectively. The right panels display for each effort η and targeted strategy s, the ratio between the fractions of the population $\mu_s(\eta)$ and $\mu_0(\eta)$ that are expected to be vaccinated when strategy s and the traditional campaign are employed, respectively. In all panels, Strategies 1, 2, 3 are depicted in blue, green, and magenta, respectively, and the intensity of the vaccination campaign is set to $\alpha = 1$.

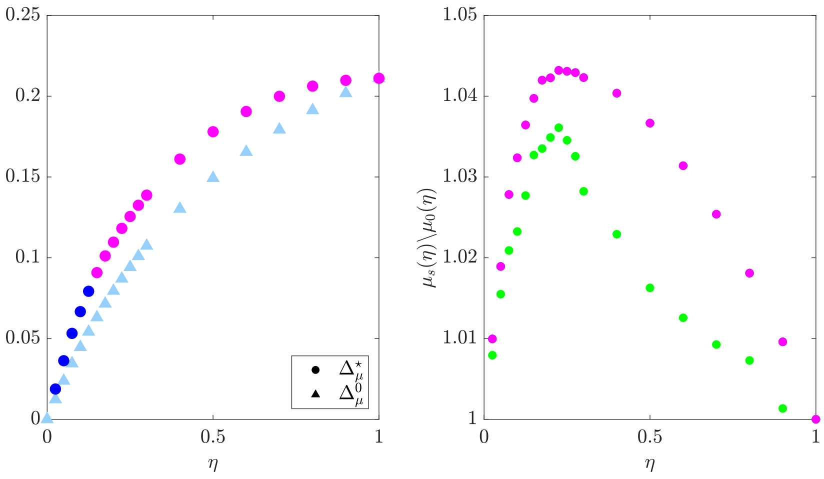

Figure S4: Comparison of targeted and traditional mass campaigns. The graph deployed is an unweighted undirected network of Facebook friendships. The number of nodes $N = 2300$, the number of edges $|\epsilon| = 96400$, the average degree $k_{av} = 83$.

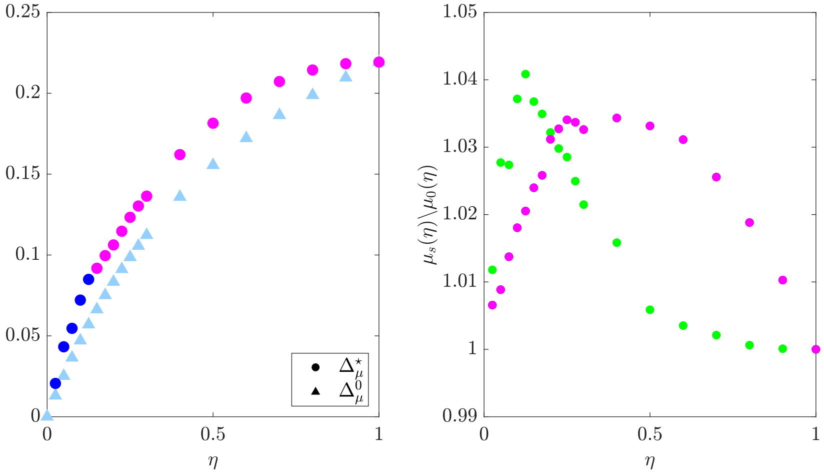

Figure S5: Comparison of targeted and traditional mass campaigns. The graph deployed is an unweighted undirected network of the friendships and family links between users of the website `http://www.hamsterster.com.`. The number of nodes $N = 2400$, the number of edges $|\epsilon| = 16600$, the average degree $k_{av} = 13$.

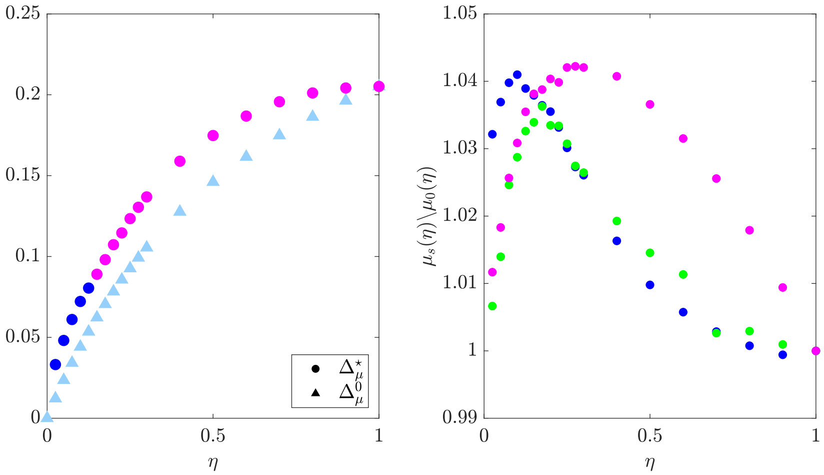

Figure S6: Comparison of targeted and traditional mass campaigns. The graph deployed is an unweighted undirected network of Facebook friendships. The number of nodes $N = 1500$, the number of edges $|\epsilon| = 33000$, the average degree $k_{av} = 43$.

S4 Supplementary Figure

In Figure S7, we report a graphical representation of the calibration procedure described in the Methods Section of the main text.

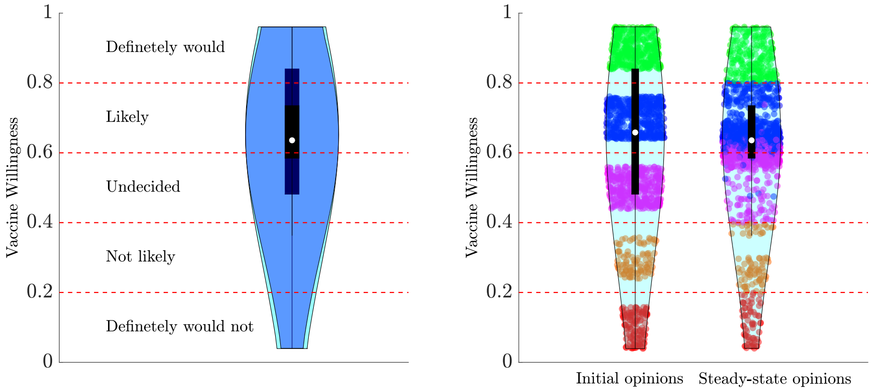

Figure S7: Violin plots of the steady-state opinion distribution from the model calibrated as described in the Methods (light blue), with that obtained from the survey data (blue), respectively. On the right, the violin plots of the initial and final opinions distribution of vaccine willingness, respectively. Data points corresponding to agents' opinions in the two endpoints are colored accordingly to their Likert score on vaccine willingness survey.

References

[1] R. A. Rossi and N. K. Ahmed, "The network data repository with interactive graph analytics and visualization," in *AAAI*, 2015. [Online]. Available: http://networkrepository.com
